# Use of Next Generation Sequencing and Synergy Susceptibility Testing in Diagnosis and Treatment of Carbapenem-Resistant *Klebsiella pneumoniae* Blood Stream Infection

**DOI:** 10.1155/2018/3295605

**Published:** 2018-01-23

**Authors:** Yuetian Yu, Fupin Hu, Cheng Zhu, Erzhen Chen, Liangjing Lu, Yuan Gao

**Affiliations:** ^1^Department of Critical Care Medicine, Ren Ji Hospital, School of Medicine, Shanghai Jiao Tong University, Shanghai 200001, China; ^2^Institute of Antibiotics, Huashan Hospital, Fudan University, Shanghai 200040, China; ^3^Department of Emergency Medicine, Rui Jin Hospital, School of Medicine, Shanghai Jiao Tong University, Shanghai 200025, China; ^4^Department of Rheumatology, Ren Ji Hospital, School of Medicine, Shanghai Jiao Tong University, Shanghai 200001, China

## Abstract

Early diagnosis and appropriate treatment for carbapenem-resistant *Klebsiella pneumoniae* (CR-*Kp*) infection is a big challenge for clinicians due to its high mortality. Every effort has been made to improve its clinical outcomes. However, treatment according to synergy susceptibility testing has never been reported in the literature. We reported a 29-year-old systemic lupus erythematosus female with CR-*Kp* blood stream infection. We highlighted the identification by next generation sequencing and treatment according to synergy susceptibility testing in the case.

## 1. Introduction

Systemic lupus erythematosus (SLE) is one of the most common autoimmune disease in Chinese population [1]. Chronic microbial colonization of the intestine, leading to exacerbations of infection when patients with immunosuppression, is the major cause of illness in SLE patients [2]. *Klebsiella pneumoniae* carbapenemases (KPCs) are plasmid-encoded carbapenem-hydrolyzing enzymes which have the potential to spread widely through gene transfer [3]. Treatment of carbapenem-resistant *Klebsiella pneumoniae* (CR-*Kp*) is difficult due to the limitation of antibiotics choice. Although next generation sequencing (NGS) is becoming increasingly important in clinical microbiology, only sporadic reports of NGS-analyzed clinical specimens have been published to date. Furthermore, synergy susceptibility testing as a new attempt to treat CR-*Kp* has never been reported in the literature.

## 2. Case Presentation

**Figure 1:**
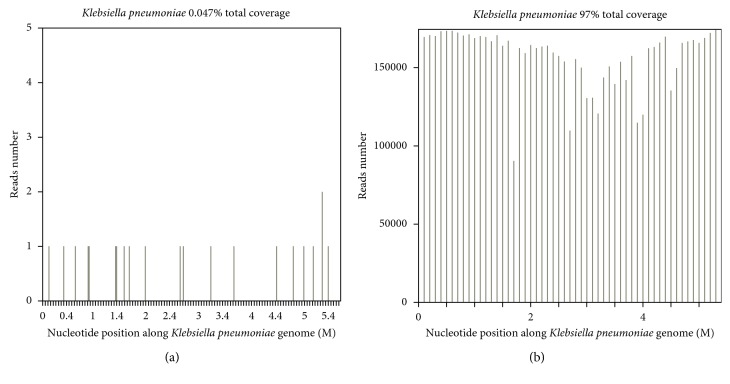
The results of next generation sequencing in blood sample. (a) Total coverage of *Klebsiella pneumoniae* in blood sample. (b) *Klebsiella pneumoniae* genome concordance.

A 29-year-old female was admitted to our intensive care unit (ICU) from the rescue room because of dyspnea for one week. Her medical history included systemic lupus erythematosus (SLE) for one year which was treated with daily oral prednisone 60 mg. Prednisone was discontinued by herself last month because she was preparing for pregnancy. On physical examination, she appeared cyanotic with fever up to 38.5°C, and moist rale could be clearly heard on the lower lobe of both sides. Chest CT scan showed diffuse interstitial pulmonary fibrosis. Acute respiratory failure was managed with protective mechanical ventilation (tidal volume 360 mL, plateau pressure 25 cm H_2_O, and positive end-expiratory pressure 15 cm H_2_O) [4]. Laboratory evaluation at the time of ICU admission revealed negative blood cultures and normal procalcitonin (0.08 ng/mL). Systemic lupus erythematosus disease activity index (SLEDAI) was 5 points which indicated that SLE was flare. Next generation sequencing (NGS) was performed to exclude infectious diseases which revealed negative in bronchoalveolar lavage fluid sample. However, *Klebsiella pneumoniae* was detected in blood sample with low coverage (0.047%) and depth (Figure 1(a)). *Klebsiella pneumoniae* genome concordance was 97% (Figure 1(b)).

Methylprednisone (40 mg q12h) was initiated prescribed to prevent progression of SLE and pulmonary fibrosis. Meropenem (1 g q8h) was instituted to treat the probable blood stream infection (BSI). Three days after admission to ICU, fever of the patient was up to 40.1°C, and procalcitonin increased to 43.7 ng/mL. Blood cultures were positive for carbapenem-resistant *Klebsiella pneumoniae* (CR-*Kp*) according to bioMérieux Vitek-2 automated system. Antimicrobial susceptibility testing was performed, and the breakpoint (susceptible, intermediate, or resistant) was determined according to *Enterobacteriaceae* M100-S27 provided by the Clinical and Laboratory Standards Institute (CLSI) standards. The minimal inhibition concentration (MIC) of meropenem was ≥16 mg/L. The antibiotic susceptibility profiling revealed that this strain of CR-*Kp* was susceptible only to tigecycline (MIC = 0.75 mg/L) and colistin (MIC = 0.25 mg/L). However, colistin was not available in the mainland China.

High-quality sequencing data were generated by removing low-quality reads, adapter contamination, and reads with a certain proportion of Ns' bases. Then, the remaining reads were aligned to Bacterial Virulence Factor Database (VFDB) using the Burrows Wheeler Alignment tool (BWA, Version 0.7.10). The coverage rate and depth of every bacterial virulence gene were calculated by Short Oligonucleotide Analysis Package (SOAP, http://soap.genomics.org.cn, Version 2.7.7). Drug resistance and virulence genes were detected, and *Klebsiella pneumoniae* carbapenemases-2 (KPC-2) was confirmed as the main (Table 1).

**Figure 2:**
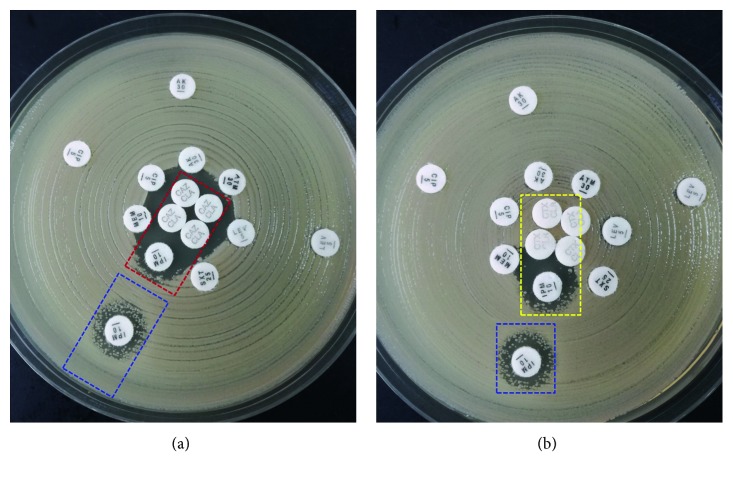
The results of synergy susceptibility testing. (a) Synergy susceptibility testing represented synergism between imipenem and ceftazidime (red square). CR-*Kp* was resistant to imipenem (blue square). (b) No synergism between imipenem and clavulanic acid (yellow square). CR-*Kp* was resistant to imipenem (blue square). CAZ, ceftazidime; IPM, imipenem; CLA, clavulanic acid.

A systematic review of the literature showed that tazobactam and clavulanic acid are partial inhibitors of KPCs and the hydrolysis of the third generation cephalosporin is relatively weak in KPCs [5]. We performed a synergy susceptibility testing based on large doses of ceftazidime-clavaminic acid and found that it represented synergism between imipenem and ceftazidime (Figures 2(a) and 2(b)). Although it was just in vitro, it might be the best therapeutic option at that time. Imipenem-cilastatin (1 g q6h) and ceftazidime (2 g q8h) combined with tigecycline (100 mg q12h) were started. Three days later, blood cultures turned negative, procalcitonin level dropped to 0.23 ng/mL, and the fever disappeared. One week later, NGS of blood sample revealed negative, and antibiotics were discontinued due to kidney injury and digestive side effects. The patient was discharged with a good health condition 10 days later, and there was no recurrence of BSI.

## 3. Discussion

KPCs are plasmid-encoded carbapenem hydrolyzing enzymes which have the potential to spread widely through gene transfer. Notable among the drugs under development against KPC are mostly derivatives of polymixin, *β*-lactamase inhibitor NXL104 with combination of oxyimino cephalosporin as well as with ceftazidime [6]. The KPCs have become endemic, and treatment options for these infections are limited because colistin and ceftazidime–avibactam are not available in the mainland China. In these patients, treating with tigecycline alone showed a higher mortality despite the in vitro susceptibility [7]. Therefore, new methods should be sought to improve the clinical outcome.

Susceptibility methods vary in terms of choice of media, inoculum preparation, antimicrobial disk content, breakpoints, and interpretation of those breakpoints around the world [8]. Even when these variables are taken into consideration, susceptibility testing of *Klebsiella pneumoniae* remains challenging given the multiple mechanisms of resistance, both intrinsic and acquired, which are frequently expressed concurrently, often at low levels [9]. The hydrolysis of the third generation cephalosporin is relatively weak in KPCs; thus, we took ceftazidime as the foundation of the synergy susceptibility testing. The in vivo experiment demonstrated synergism between imipenem and ceftazidime and achieved a better therapeutic effect in this patient. However, disk diffusion testing does not correlate well with MIC results, and no cutoff value was defined about synergy susceptibility testing. Maybe it is just a new attempt and exploration, and we still need more studies with large samples to prove the real effect.

BSI remains one of the major challenges in critical patients, which, if not treated promptly, will lead to septic shock and multiple organ failure [10]. Due to the lack of timely diagnostic approaches with sufficient sensitivity, mortality of these patients is still unacceptably high. Blood culture-based diagnostic procedures represent the standard of care, although they are associated with relevant limitations [11]. Two caveats should be born in mind in interpreting the blood culture results: (1) culture-based diagnostic procedures often reveal false negative results due to the administration of an empiric antibiotic therapy, and (2) growth of causative pathogen often takes up to 5 days for results to become available [12]. Therefore, an empirical diagnosis of the causative microorganism is critical to improve outcome of BSI.

NGS-based diagnostic testing might offer several advantages over blood culture. First, it is an open platform, providing the opportunity to detect bacterial, fungal, and viral pathogens in a single assay. Second, it is quantitative through counting of sequence reads and calculation of statistical significance. Third, it is unbiased and untargeted, so it benefits from any DNA sequence information within patient specimens, potentially delivering higher sensitivity and specificity [13, 14]. However, although NGS is becoming increasingly important in clinical microbiology, only sporadic reports of NGS-analyzed clinical specimens have been published to date. Furthermore, it remains largely unknown whether treatment strategy based on NGS is able to shorten the course of antibiotics which is worth further discussing.

In summary, the KPCs have become endemic, and treatment options for these infections are limited. Synergy susceptibility testing is a new attempt and exploration, which still needs more studies with large samples to prove the true effect. NGS analyzes the circulating cell-free DNA (cfDNA) from plasma samples of septic patients and has higher sensitivity and specificity. Combining NGS with synergy susceptibility testing may improve the outcome of CR-*Kp* BSI patients.

## Figures and Tables

**Table 1:** The top five drug resistant and virulence genes of this strain *Klebsiella pneumoniae*.

Gene names	Protein	Percentage	Depth
*Drug resistance gene*			
*KPC-2*	Carbapenem-hydrolyzing class A beta-lactamase, KPC-2	100	230
*aadA2*	ANT(3″)-Ia family aminoglycoside nucleotidyltransferase	100	220
*CTX-M-24*	Class A extended-spectrum beta-lactamase, CTX-M-24	100	180
*QnrS1*	Quinolone resistance pentapeptide repeat protein, QnrS1	100	180
*dfrA12*	Trimethoprim-resistant dihydrofolate reductase, DfrA12	100	180
*Virulence gene*			
*bla*	Methylmalonic aciduria type A protein	100	1400
*fepA*	Ferrienterobactin receptor	100	810
*mrkD*	Fimbria adhesin protein	100	810
*iutA*	Putative ferric siderophore receptor	100	650
*manB*	Mannosidase, beta A, lysosomal-like	100	590

Annotation of drug resistance gene: *KPC-2*, carbapenem-hydrolyzing class A; *aadA2*, aminoglycoside resistance; *CTX-M-24*, beta-lactamase class A; *QnrS1*, quinolone resistance pentapeptide repeat protein; *dfrA12*, dihydrofolate reductase (trimethoprim resistance protein).

## Abbreviations

CR-*Kp*: Carbapenem-resistant *Klebsiella pneumoniae*
NGS: Next generation sequencing
MIC: Minimal inhibition concentration
KPCs: *Klebsiella pneumoniae* carbapenemases.
